# Evolution of the World Health Organization’s programmatic actions to control diarrheal diseases

**DOI:** 10.7189/jogh.09.020802

**Published:** 2019-12

**Authors:** Cathy Wolfheim, Olivier Fontaine, Michael Merson

**Affiliations:** 1 World Health Organization, Geneva, Switzerland (retired); 2 UNICEF, Geneva, Switzerland (retired); 3 Duke Global Health Institute, Duke University, Durham, North Carolina, USA

## Abstract

The Program for the Control of Diarrheal Diseases (CDD) of the World Health Organization (WHO) was created in 1978, the year the Health for All Strategy was launched at the Alma Ata International Conference on Primary Health Care. CDD quickly became one of the pillars of this strategy, with its primary goal of reducing diarrhea-associated mortality among infants and young children in developing countries. WHO expanded the previous cholera-focused unit into one that addressed all diarrheal diseases, and uniquely combined support to research and to national CDD Programs. We describe the history of the Program, summarize the results of the research it supported, and illustrate the outcome of the Program’s control efforts at country and global levels. We then relate the subsequent evolution of the Program to an approach that was more technically broad and programmatically narrow and describe how this affected diarrheal diseases-related activities globally and in countries.

The recent history of child survival programs best starts in 1979 when the United Nations Educational, Scientific and Cultural Organization (UNESCO) proclaimed the International Year of the Child to draw attention to problems that affected children throughout the world. Soon thereafter, in 1982, the United Nations Children’s Fund (UNICEF) launched the Child Survival Revolution that proposed to reduce childhood mortality and morbidity significantly through the use of four simple approaches: growth monitoring, oral rehydration therapy, breastfeeding and immunization. The year 1989 saw another milestone, when the United Nations (UN) General Assembly adopted the Convention on the Rights of the Child, asserting on behalf of children that their basic rights to health and education should ultimately be guaranteed by the State. Continuing this movement, a lengthy set of goals was established in 1990 at the World Summit for Children, to be achieved by 2000, that included a one-third reduction in under-five mortality rate and a 50% reduction in deaths due to diarrhea.

In 2001, the General Assembly adopted eight Millennium Development Goals (MDGs) [1], one of which (MDG4) aimed to reduce under-five mortality by two-thirds between 1990 and 2015. Significant progress was achieved: the global under-five mortality rate had declined by more than half by 2015 [2], at which time the Sustainable Development Goals (SDGs) were adopted [3]. SDG 3: Good Health and Well-being, calls for ending preventable deaths of newborns and children under five, with targets of reducing neonatal mortality to 12 per 1000 live births and under-five mortality to 25 per 1000 live births by 2030.

Throughout this time period, various global programs sought to convert the above commitments into results. In the 1970s and 1980s in particular, national health ministries moved quickly and steadily to roll out primary health care interventions and increase the reach of health services. Interventions were chosen based on their potential impact and feasibility, resulting in what is known as selective primary health care. Research on the best means of implementation helped the design and redesign of programs. The consistent decrease in mortality generated a lot of interest and investment, and primary health care approaches continued to flourish.

It was within this stimulating context that WHO, with the strong support of its Director-General, created the Program for the Control of Diarrheal Diseases (CDD). As one of the pillars of the Health for All Strategy launched at the Alma Ata Conference in 1978, the CDD Program’s primary goal was to reduce diarrhea-associated mortality among infants and young children in developing countries [4]. It departed from the standard WHO operational model at that time, first by expanding the former cholera-focused unit into a Program that addressed all diarrheal diseases, and second by combining research with significant support to country implementation activities. The present paper presents the history of that Program, its subsequent evolution from one that was technically focused and programmatically broad to an approach that was technically broader and programmatically narrow, and the subsequent impact of this evolution on diarrheal control activities globally and in countries.

## Early history of CDD program development

Up to the late 1970s, the standard recommendation for treating diarrheal disease was to use antidiarrheal drugs and encourage patients to refrain from eating and drinking for 24 to 48 hours. Dehydrated patients were given IV therapy if available. It was commonly accepted that socioeconomic development, particularly water and sanitation, was the key to reducing the incidence of diarrhea. This meant a long wait for many places, while between 2.2 and 3.1 million children were dying annually from diarrhea [5].

Based on the results of studies treating cholera among adults and children in Bangladesh and India in the mid-1970s [6-8], oral rehydration therapy (ORT) was adopted as standard treatment of diarrhea for those patients with mild to moderate dehydration and who were able to drink. Oral rehydration salts (ORS) solution was added to the WHO list of essential drugs, and in 1978 WHO established the global CDD Program [4]. In a structure that was at that time unique for WHO, the Program combined two distinct and interlinked approaches: 1) research to discover improved and new “tools” for future application, and 2) support to national CDD programs to apply existing knowledge about the treatment and prevention of diarrhea. Joining these components into a single program ensured that research findings were rapidly incorporated into program guidelines.

The Program made a carefully considered decision to focus on reducing mortality by promoting case management. Clinical guidelines were developed and corresponding treatment plans were created for use in health facilities and by community health workers [9] (**Box 1**). ORT was one of the low-cost health technologies singled out in the UNICEF Child Survival Revolution. This led to significant collaboration between WHO and UNICEF, including the presence of a CDD focal point in UNICEF headquarters, shared staff positions between UNICEF and WHO, joint support to local ORS production and to behavior change communication, and financial and technical support to countries. Bilateral agencies, especially the United States Agency for International Development (USAID), also made CDD a priority and provided substantial support to country programs.

Box 1 **Clinical management of diarrhea: three main points**

The linchpin of the CDD Program was the proper management of diarrheal disease, with a simple three-part approach (7):

- Prevent or treat dehydration with ORS solution (IV only when indicated),

- Continue feeding and breastfeeding during the diarrhea episode,

- Use antibiotics only in cases of bloody diarrhea.

## Support to research

The CDD research agenda evolved significantly between 1980 and 1995. Most of the initial projects examined the etiology and epidemiology of childhood diarrhea. Early studies showed that regardless of the setting, most cases were caused by essentially five organisms: rotavirus, *E coli*, *Shigella*, *Campylobacter jejuni*, and *Cryptosporidium* [10].

Subsequent research was oriented to the development and evaluation of interventions for implementation. The list below summarizes those studies considered to be most important because they led to new or improved interventions, both for treatment and for prevention.

- ORS formulations – The original ORS solution had the following formula: 111mmol/L glucose, 90 mmol/L sodium, 30 mml/L bicarbonate, 20 mmol/L potassium, 80 mmol/L chloride, total osmolarity 331 mmol/L. Studies in the 1980s showed that replacing 30mmol/L sodium bicarbonate with 10mmol/L tri-sodium citrate resulted in a more stable, and therefore more simply and cheaply packaged ORS packet [11,12]. In the 1990s, more than 50 studies compared the standard citrate ORS with experimental formulations (based on glycine, L-alanine, L-glutamine, maltodextrin, rice and other cereals) that might enhance absorption. None was found to have any advantage over the standard citrate ORS [13] (**Box 2**) [9]. It was later shown that reducing the osmolarity of ORS (lower sodium and glucose concentrations) reduced stool output, vomiting, and more importantly the need for unplanned IV therapy in children. A revised lower osmolarity ORS solution was adopted in 2002 as the recommended WHO formula [14].
  Box 2 **Early recommendations on home fluids**
  - For many years, home fluids were recommended for treating children who had diarrhea but no signs of dehydration. These solutions needed to contain salt, be given frequently to the child, and be acceptable to caregivers. The early recommendation to make and use “Sugar Salt Solution”, a mix of 3g/L of table salt and 18g/L of common sugar, was later abandoned because the recipe was often forgotten or mixed incorrectly, or because too little solution was given to the child to drink.
- Persistent diarrhea – Up to 20% of diarrhea episodes last for 14 days or more, and these “persistent” cases have a high risk of mortality [15]. Studies showed that patients hospitalized with persistent diarrhea could be treated successfully with ORS and a reduced lactose diet based on locally available and inexpensive foods [16].
- Antidiarrheal drugs - Concern about the effects of widespread use of drugs to stop diarrhea prompted the CDD Program to conduct a review of all common anti-diarrheal drugs. The review, published in 1990, concluded that none of these drugs were sufficiently efficacious or free from adverse side effects and thus they should not be used in the routine management of diarrhea [17].
- Antibiotics – Evidence that approximately 10% of diarrheal episodes in children under five years of age have blood in the stool led the Program to support research on treatment guidelines for bloody diarrhea. This showed that use of an antimicrobial known to be effective against *Shigella*, the most important cause of dysentery, leads to clinical improvement within two days [18,19]. Research also demonstrated that there was no benefit to the routine use of gentamicin or cotrimoxazole for persistent diarrhea.
- Preventive measures – There was an abundance of potential interventions for preventing diarrhea. To determine which would be the most useful to promote, the CDD Program commissioned in-depth reviews of the potential effectiveness, feasibility, and cost of 18 such interventions [20]. The most promising interventions, defined as those with potentially high effectiveness and feasibility, included the promotion of breastfeeding, the improvement of complementary feeding, and measles immunization. As a result of the reviews the first two interventions were included as primary CDD program strategies (measles immunization was included in the WHO Expanded Program on Immunization (EPI)).
- Breastfeeding – Studies confirmed that infants who are exclusively breastfed for up to six months have fewer diarrheal episodes and a lower risk of persistent and severe diarrhea [21,22]. Moreover, they gained adequate weight, and introducing complementary foods before this age did not increase weight gain. Further research validated that feeding bottles were often highly contaminated with fecal bacteria and thus increased the risk of infection. Based on these findings, the CDD Program recommended that 1) infants be exclusively breastfed for the first six months of life, and 2) the use of feeding bottles and the giving of other fluids such as teas or water during this period should be discouraged. Strong evidence that breastfeeding counselling by health personnel was a highly effective intervention led the Program to develop a training course on this skill. Subsequent research concluded that training in lactation management and effective counselling of mothers can increase exclusive breastfeeding [23,24].
- Complementary feeding – After malnutrition was shown to be associated with increased severity of and mortality from diarrhea, pneumonia and measles, studies were carried out to address improved complementary feeding [25,26]. Early studies had indicated that impaired growth in the 6- to 11-month age period reflected the effects of repeated illness and inadequate energy intake [27], and that the latter may be due to low frequency of meals, small size of feeds, and low energy density of food. These results led to interventions to address specific local problems, for example encouraging greater frequency of feeding in Guatemala [28] and promoting more energy-dense foods in Peru [29], as well as a general recommendation to feed a sick child more frequently [25].
- Water and sanitation – Research related to water, sanitation and hygiene in the prevention of diarrhea showed that the availability of water (quantity) is more important than its quality [30,31]. It was also found that three water-related hygiene behaviors significantly affected the incidence of diarrhea: handwashing, sanitary disposal of feces, and keeping drinking water free from fecal contamination. Of these, handwashing was found to have the most significant effect [32]. This research influenced the content of communication messages related to water use.
- Vitamin A supplementation – Studies found that vitamin A supplementation reduced the incidence and severity of diarrhea, especially during highly purging episodes [33].
- Zinc supplementation – Results from randomized controlled trials showed that supplementation with zinc reduced the incidence and severity of diarrhea for several months following treatment. This led to the recommendation in 2004 that children with diarrhea should receive zinc supplements for up to 15 days [14]. Subsequent studies revealed that children with acute diarrhea treated at the peripheral level with zinc and ORS coupled with caregiver education had 1) a reduction in the prevalence of diarrhea and pneumonia and in hospitalization for diarrhea, pneumonia and all causes, 2) easier access to diarrhea case management within the village itself, and 3) reduced use of unwarranted oral and injectable drugs for diarrhea [34,35]. ORS use increased substantially in the intervention areas and was not compromised by the concurrent administration of zinc during diarrhea [34,35].

## Support to national program implementation

These research findings provided a strong foundation on which the CDD Program was able to base its support to governments in implementing innovative and comprehensive national CDD programs. Support to country implementation was influenced by that provided by EPI, which had been initiated a few years earlier. Countries were encouraged to designate national and where appropriate sub-national focal points (program managers), to establish a national CDD program with an implementation plan, and to include a specific line for CDD activities in national health budgets. The following activities were given priority:

### Training on (clinical) case management

Training in the clinical management of diarrhea took various forms, starting with the establishment of diarrhea training units in health facilities and expanding to cover training institutions and distance learning.

Diarrhea training units - In the early days of the Program, the quality of courses to teach health workers how to manage diarrhea relied on the pre-existing skills of trainers. A large proportion of training took place in classroom situations, with limited opportunity for hands-on practice. As a result, many trainees acquired knowledge but the quality of their performance was often inadequate. To address this problem and stimulate centers of excellence in diarrhea case management training, the Program developed a package called “The Diarrhoea Training Unit (DTU): Director’s guide and training materials” in 1986. An evaluation carried out one year later showed that 156 DTUs, including 70 that were also Acute Respiratory Infection (ARI) Training Units, were considered functional based on a list of established criteria. Many served multiple functions, since they were located primarily in teaching institutions.

After ascertaining that it was not feasible to create and maintain enough DTUs to cover all the training needs of countries, the Program developed “Guidelines for conducting clinical training courses at health centers and small hospitals” in 1990. These guidelines were used, with some revisions in 1992, as the basis for in-service (on-the-job) training of health workers. Overall, by the end of 1995 over 7300 courses had been held and more than 570 000 health workers trained. This represented an estimated 38% of health workers who regularly treated cases of child diarrhea. In 1987, the Program also undertook steps to formally monitor the quality of this training.

Pre-service training - Considerable effort also went towards improving teaching in medical schools and other training institutions for health professionals. A package of materials entitled “Strengthening the teaching of diarrheal diseases in medical schools” became available in 1992-1993, and a similar set of materials was developed for nursing and other training institutions in 1994-1995. By the end of 1995, staff from 152 medical schools in 35 countries had participated in workshops to plan how better to teach diarrhea management to medical students. A subsequent review of progress in three countries (Bangladesh, Ethiopia, and Myanmar) confirmed that the schools assessed were dedicating more time to teaching about diarrheal diseases, using the WHO case management guidelines and wall chart to teach students, and placing more emphasis on clinical instruction and interactive teaching.

Distance learning - To further extend access to training, the Program developed a course for distance learning, “Clinical skills: a self-instructional course”. This course allowed health workers to learn about diarrhea case management in their own health facility with support through correspondence or visits from a tutor. The course became available in 1993, but experience with its use was very limited due to resource constraints.

The CDD Program worked closely with the publishers of major textbooks of pediatrics to ensure that revisions and updates included all relevant recommendations on managing diarrheal diseases. By 1995, the text of chapters on diarrheal diseases in all major textbooks of pediatrics was in line with WHO recommendations.

### Training on program management

The Program developed and promoted a course to provide national CDD program managers with the skills needed to plan, oversee, monitor and evaluate national and sub-national CDD activities. First published in 1980, the materials were revised in 1988 to respond to changes in global policy and the needs of countries. Training modules covered how to define or revise national policy, set national targets, plan and monitor activities, and evaluate program performance in order to influence future planning. Courses were held at global, intercountry, and country levels.

In 1985, a mid-level management “Supervisory skills” course was developed and promoted. This course was intended to strengthen the supervision of health facilities as well as the capacity of health workers to carry out case management.

### Local production of ORS

The first guidelines for producing ORS were issued in 1980. The CDD Program devoted considerable attention to the development or strengthening of pharmaceutical facilities mostly in low and middle income countries to produce ORS of acceptable quality, and ensuring adequate availability of ORS packets. Production guidelines were revised and re-issued in 1985, and again in 2005, when the lower osmolarity ORS became the WHO recommended formula.

**Figure 1:**
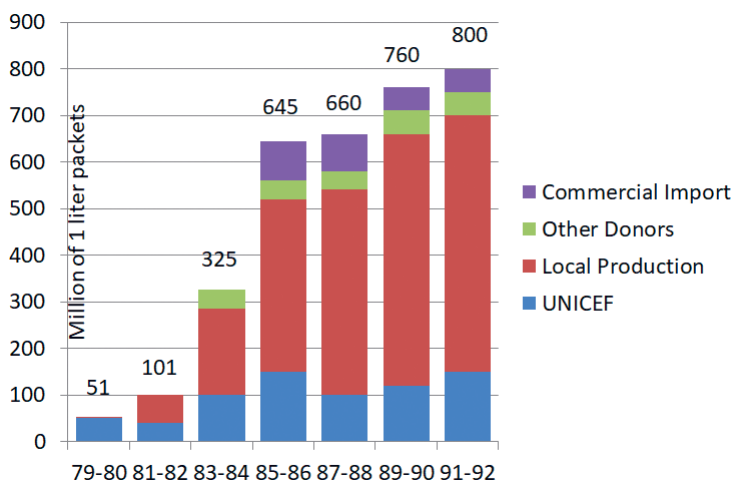
ORS supply in 60 developing countries (1979-1992). Source: The Evolution of Diarrhoeal and Acute Respiratory Disease Control at WHO [11].

Production was carefully monitored. In 1993, approximately 60 developing countries were producing ORS of satisfactory quality. The evolution of the proportion of locally-produced ORS is shown in **Figure 1**. No information on ORS production (aside from UNICEF production) was collected after 1993.

### Behavior change communication

Evaluations in the late 1980s revealed that health workers performed relatively poorly on advising caregivers on home care for children with diarrhea. This element was strengthened, first by establishing three rules of home care: (i) Give the child more to drink than usual, (ii) Give the child soft mashed foods and continue breastfeeding, and (iii) Take the child to a health worker if danger signs appear (child unable to drink or breastfeed, child vomits everything, child has convulsions, child is lethargic or unconscious). A session on “Advising mothers on management of diarrhea in the home – a guide for health workers” was developed and incorporated into case management training. Health workers were taught to follow a simple process: *Ask – Praise – Advise – Check understanding*; the same process was subsequently integrated into training courses on CDD.

Communication beyond the health facility was a critical and novel CDD program component, and family knowledge and practices gradually changed in response to intensive efforts. Sound communication strategies, often in collaboration with UNICEF or with specialized communication agencies, involved the mobilization of communities and households, and contributed to increasing the use of ORT and changing behavior. Where these strategies were used, for example in Brazil, Egypt, and The Gambia - the positive changes were significant [36-38]. Unfortunately, most countries did not give adequate attention and resources to communication, and communication expertise was often not used.

CDD programs offered the context for ground-breaking research on communication strategies and activities, which in turn provided the basis for communication in other programs [39]. Behavior change communication became an accepted part of program implementation and standard operating procedure for, most notably, HIV/AIDS, malaria, family planning, and vaccination programs.

## Monitoring and evaluation

Monitoring and evaluation (M&E) were fundamental to the success of the CDD Program (**Table 1**). Health facility surveys, developed in 1988, assessed the quality of health worker performance and identified strengths and weaknesses in health system supports; household surveys, developed in 1984, evaluated family knowledge and practices including care seeking; and program reviews, developed in 1986, identified and proposed solutions to problems in implementation. These activities were conducted with regular periodicity and data were aggregated at regional and global levels to track trends and common problems. Data were also a vital part of WHO annual reporting, so that countries, partners and donors could follow the successes and challenges of implementation. Constant and continued visibility, coupled with pressure to improve implementation, resulted in continued expansion of activities and the control and improvement of quality.

**Table 1:** Milestones in the development of CDD evaluation tools

Year	Program management tools	Household-based tools	Health facility-based tools
**1980**	CDD Program Management training course, with module on evaluation		
**1984**		Guidelines for a sample survey of diarrheal disease morbidity, mortality and treatment rates	
**1986**	Guidelines for conducting comprehensive review of a diarrheal disease control program	Household survey manual: diarrhea case management, morbidity and mortality	
**1988**			Health facility survey manual: diarrhea case management, morbidity and mortality
**1990**	Guidelines for conducting a focused program review	A manual for the measurement of childhood mortality with simple surveys – UNICEF/WHO/LSHTM	
**1996**	Guidelines for conducting a short program review		

The regular collection of information on CDD program indicators continued up to about 1996. Some of the principal achievements at that time included:

- 425 CDD household surveys conducted until 1996,
- 45 Health facility surveys conducted until 1996,
- 120 program reviews conducted until 1996,
- 80 countries had operational national CDD programs,
- 720 people in 24 countries had been trained in CDD program management,
- 980 people in 22 countries had been trained in mid-level management,
- 520 trainers were trained to teach case management,
- 80 countries had health education materials available,
- 175 program evaluations had been conducted,
- 50% of cases of child diarrhea had access to ORT (increased fluids plus continued feeding),
- 37.5% of diarrhea cases received ORT.

By 1995, it was estimated that 80% of diarrhea cases had access to ORS and 34% of cases were using ORT. The use of unnecessary IV drips almost completely disappeared in the main hospitals in developing countries. However, only 24% of caretakers had adequate knowledge of the three rules of home care, down from 32% in 1992.

## A unified global community

A crucial contributing factor to the strength and success of CDD was the unanimity of the global health community. It was firmly united around the CDD Program, its objectives, its support to national programs, and the indicators it used to monitor their progress. Leadership was strong and effective. WHO was respected for its strong technical and programmatic guidance. The Executive Director of UNICEF from 1980 – 1995, the visionary James Grant, would often pull an ORS packet from his pocket in any setting he was in. The International Conference on ORT (ICORT), held in 1983 and 1985, under the auspices of USAID and in cooperation with WHO, UNICEF and the International Center for Diarrheal Diseases Research, Bangladesh (ICDDR, B), was exceptionally well attended. Newsletters such as Diarrhea Dialogue were published regularly by AHRTAG, a UK based nongovernmental organization, with updates on technical and programmatic advances.

**Figure 2:**
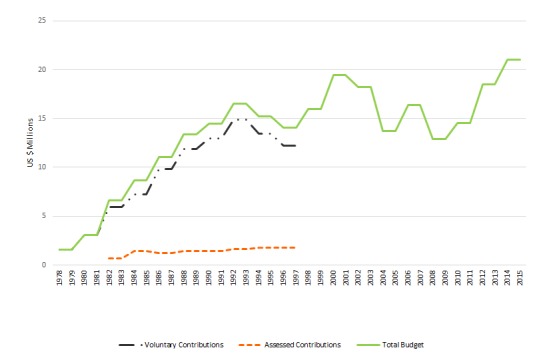
WHO Financial Resources for Diarrheal-related Activities from Assessed Contributions (WHO core budget), Voluntary Contributions, and Total Budget (1978-2015). Figures after 2012 are projected budget only and do not reflect funds received. Source: WHO (1979 to 2015): 1978-1989 – Diarrheal Diseases Control Program (CDD), 1990-1995 – Division of Diarrheal and Acute Respiratory Diseases Control (CDR), 1996-1998 – Department of Child Health and Development (CHD), 1999-2009 – Department of Child and Adolescent Health and Development (CAH), 2010 – Present – Department of Maternal, Newborn, Child and Adolescent Health and Development (MCA).

As shown in **Figure 2**, donor support to the WHO programs responsible for diarrhea control grew considerably from 1978 to 1992, and was greatest during the period when the CDD program had its own specific budget. After the early 1990s, while the mandate of the department responsible for CDD activities expanded considerably, as described below, funding levelled off. The sizeable increase in the mandate of the WHO Department responsible for CDD activities was not matched with an increase in funding or of staffing.

## Beyond the CDD Program

Between 1990 and 2010, successive reorganizations within WHO resulted in an enormous transformation of the Diarrhea Disease Control Program (Table S1 in **Online Supplementary Document**). Global and national CDD program activities had resulted in a significant decline in the number of diarrhea-related deaths, resulting in a proportional increase in child mortality due to pneumonia. In 1990, the WHO CDD Program merged with the parallel Acute Respiratory Infections (ARI) Program to create the WHO Division of Diarrheal and Acute Respiratory Disease Control (CDR), with the aim of reducing under-five mortality due to these two conditions. Available tools for both programs were merged wherever possible resulting in combined diarrhea-pneumonia training courses for clinical management and program management and materials for communication (including applied anthropology tools such as identifying local terms) and M&E.

In 1996, CDR metamorphosed into the WHO Department of Child Health and Development (CHD), with an expanded mandate that included the main causes of child mortality plus child development. In 1998, adolescent health was included and the Department was renamed Child and Adolescent Health (CAH). In 2010, maternal health and newborn health were added and department renamed again as Maternal, Newborn, Child and Adolescent Health (MCA). Thus, although the responsibility for CDD remained largely within the same WHO programmatic structure, it gradually evolved from being the principal focus of a single program to being one focus of a much broader program. A main reason for these changes was the observation that sick children often had a set of symptoms and signs that could indicate a number of conditions, and thus it was more effective and logical to provide health workers with the skills to assess and treat the child as a whole rather than for a single specific disease.

In response, WHO in collaboration with UNICEF, used the clinical and programmatic experience gained largely from CDD to develop Integrated Management of Childhood Illness (IMCI). Officially launched in 1997, IMCI aimed to increase coverage of a selected set of evidence-based, high-impact interventions, taking an integrated approach to promoting, preventing, and treating pneumonia, diarrhea, malaria, measles and malnutrition. IMCI targeted countries with child mortality rate higher than 40 per 1000 live births and was organized around three components: to improve 1) health worker skills, 2) health systems, and 3) family and community practices.

Early IMCI activities focused on developing and implementing a clinical training course for first-level health workers. This course lasted 11 days and, patterned after CDD training, devoted a large percentage of time to hands-on practice. Countries that found the 11-day period too demanding developed shorter courses with less time for clinical practice. For actions at the family and community level, WHO and UNICEF defined a set of evidence-based behaviors, and various agencies developed frameworks for promoting these: early and exclusive breastfeeding, adequate complementary feeding, micronutrients, immunization, use of bednets, social and mental development, feeding and fluids for sick children, home treatment for infections, appropriate care-seeking, follow health worker recommendations, hygiene, antenatal care, prevent child abuse, prevent HIV and care for affected children, male participation in child care, prevent and treat injuries. The need to increase access to curative interventions led to the decision to include lay community health workers (CHW) in the provision of care. In 2011, a package for training CHWs these skills was finalized by WHO and adopted as the gold standard by an interagency integrated Community Case Management task force (iCCM) [40].

In contrast to the CDD approach, which focused on providing support to national CDD programs, IMCI was considered and promoted as a strategy. Consequently, there was by design, no direct support for global or national IMCI programs, as there had been for CDD. For the most part, support for national CDD program activities came to an abrupt end, a move that was neither understood nor appreciated by countries and partners. However, WHO did support countries to adapt IMCI guidelines, plan interventions, train health workers, and undertake other specific activities based on country needs. In 2009, WHO produced the guidelines “Managing programs to improve child health” [41] to help countries in their planning and implementing specific interventions, including but not limited to IMCI. Other tools included guides for adapting IMCI to the local situation, for developing local terms, for planning and for conducting reviews of national child health programs. Over time, with the reduction in deaths of children aged 1 month to 5 years, the relative importance of newborn mortality increased. Thus, newborn survival and health were added as objectives, and in some countries IMCI was revised to become Integrated Management of Neonatal and Childhood Illness (IMNCI).

The progressively diluted leadership from WHO and UNICEF, combined with inadequate and unsustained funding, led to a loss of collaboration and synergy between the three components of IMCI, as well as a lack of focus and momentum. UNICEF participated in but did not lead on the community component, as had been proposed at the launch of the strategy in 1997. The international community was divided over the utility of IMCI, with some partners even proclaiming “IMCI is dead”.

IMCI has undergone a number of formal global evaluations and reviews, most notably the Multi-Country Evaluation, the Analytic Review [42-44], a Cochrane Review [40] and most recently a WHO/UNICEF Strategic Review [45]. There have also been regional and individual country reviews. All these exercises revealed limitations in the coverage and quality of the IMCI interventions. They also suggested that the lack of explicit emphasis on equity, community engagement, and linkages to other sectors (such as water and sanitation) limited the impact on child mortality.

The 2016 WHO/UNICEF Strategic Review [45] did find that there was near-universal adoption of the strategy by target countries, at least at the policy level. More importantly, it revealed that IMCI was associated with a 15% reduction in child mortality when activities were implemented in both health facilities and communities, and that 23 countries (26% of those participating in the review) were implementing iCCM in at least three quarters of their districts. These findings are supported by other assessments of iCCM which highlighted increasing policy adoption and implementation, particularly in Africa and South-East Asia. At the same time, the 2016 review identified deficiencies that need to be addressed. These included the need for greater clarity regarding program strategies, more evidence of the impact of programs, increased financial resources, and much greater accountability by WHO and UNICEF for the success of IMCI.

## CONCLUSIONS

A paradigm shift took place when the WHO CDD program was replaced by the WHO CAH department, and later when CDD program activities were subsumed under the IMCI strategy. The very specific global and country-level objectives and targets of CDD were either done away with or became less important to monitor. The systematic, stepwise process used by CDD to guide countries to plan, implement, monitor, and evaluate program activities was lost. National CDD focal points were no longer in place and CDD budget lines were rarely included in ministry of health budgets. The shift from a focused CDD program to a broader IMCI strategy resulted in diffused responsibility and a lack of accountability for results. Furthermore, the continuum of health services provided at the health facility and in the community weakened considerably.

CDD’s encouragement, programmatic guidance, and accountability for results were replaced by a culture of “keeping child health on the agenda” and “waving the flag”. Lacking the regular monitoring of a few crucial indicators as had been done by CDD, as well as of the quality and level of implementation and coverage, IMCI is not likely to result in the mortality reductions called for in the SDGs.

It is also important to mention that concurrent to the shift from CDD to a broader MCH focus, many parallel initiatives were coming to light. These included the rise in the HIV pandemic, and the increased focus on malaria that generated global responses such as the Global Fund to fight AIDS, TB and malaria. Such initiatives competed with child diarrhea for global attention and funding, and influenced the global conversation on public health.

Somewhat related to this, we have noted that both WHO and UNICEF have recently called for a redefined, broader child health strategy [46]. We hope such an effort will take into account the great strengths of the CDD Program’s approaches, including the:

- linkages between service provision, research and evaluation;
- focus on a limited number of cost-feasible interventions;
- sound program management, high quality training, a program structure that offered strong technical support, at global and country levels; and
- sustained monitoring and evaluation of precise indicators and targets.

Accountability by the lead organizations to ensure success of the strategy and its individual components at a global level will be essential, if we are to continue to see the impressive decline that was steadily observed in diarrheal mortality [5].

## Additional material

Online Supplementary Document
