# Supplementary material for: Evolution of the World Health Organization’s programmatic actions to control diarrheal diseases
Source: J Glob Health. 2019 Oct 22;9(2):020802. doi: 10.7189/jogh.09.020802 (PMC6816052; doi:10.7189/jogh.09.020802)
Supplement: Online Supplementary Document [file jogh-09-020802-s001.pdf]

Table S1: Timeline of the evolution of CDD-related programs in WHO, 1978 – 2015

Date	Organizational event	Milestones	Objectives
**1978**	**Program for the Control of Diarrheal Diseases (CDD) established**		**To reduce under-five mortality due to diarrhea**
1980		-Training tool to help establish national CDD programmes<br>-ORS standards	
1981		-Guidelines for planning and evaluating CDD national programme activities	
1983		-Tool to strengthen supervision of health facilities and case management	
1985		-Guidance for countries to become self-sufficient in the production of ORS	
1988		-Case management of ARI added to supervisory skills training tool, compatible with CDD	
1989		-Major CDD programme components and strategies adopted by the ARI Programme to allow greater collaboration	
**1990**	**CDD and ARI merged to form the Division of Diarrhoea and Acute Respiratory Disease Control (CDR)**		**To reduce under-five mortality due to diarrhea and acute respiratory infections**
1991		-Materials revised to support joint CDD and ARI programme management training	

1992		-Introduction of a package of materials for medical schools: *Strengthening the teaching of diarrhoeal diseases in medical schools*	
1993		-Introduction of distance learning material for CDD and ARI: *Clinical skills: a self-instructional course*<br><br>-Development of a *Guide for improving treatment practices of pharmacists and other drug sellers*	
1994		-Introduction of training materials for nursing schools	
1992-1996	**WHO and UNICEF develop IMCI to address major causes of child mortality**	-11-day clinical training course on IMCI	To address more effectively the five main causes of under five deaths: diarrhoea, acute respiratory infections, malaria, measles and malnutrition
**1996**	**CDR becomes a new Division named Division of Child Health and Development (CHD)**		To address child morbidity, mortality and development
1997		-Official launch of IMCI in Santo Domingo (WHO and UNICEF)	
1998		-Drug supply management course<br><br>-IMCI follow up after training course<br><br>-UN interagency working group on household and community IMCI, with UNICEF leadership	
1999	**Health and development of adolescents are added to the responsibilities of CHD, to create the Department of Child and Adolescent Health and Development (CAH)**		Objectives added to reduce mortality and morbidity and improve development of adolescents

2000		-Management of children with severe infection and severe malnutrition<br><br>-IMCI planning guide	
2002		-IMCI Adaptation guide	
2003		-Health facility survey for quality of care	
2005		-Pocket book of hospital care for children<br><br>-Community IMCI briefing package<br><br>-Guide for educating health care providers	
2006		-Systematic review of effectiveness of shortening IMCI training<br><br>-IMCI complementary course on HIV/AIDS<br><br>-Maternal and child health delivery channel survey	
2008		-IMCI computerized adaptation and training tool (ICATT)<br><br>-Establishment of interagency iCCM task force	
2009		-Managing programme to improve child health<br><br>-WHO/UNICEF joint statement on home visits for newborn care	
2011	**Maternal and newborn health are added to the responsibilities of CAH to create the Department of Maternal, Newborn, Child and Adolescent Health**	-Global Action Plan for Pneumonia and Diarrhoea (GAPP-D)	Objective added: To reduce maternal mortality

	**(MCA)**		
2012		-IMCI generic online training course<br>-WHO/UNICEF joint statement on iCCM	
2014		-IMCI chart booklet technical update<br>-IMCI distance learning course<br>-Caring for newborn and children in the community, adaptation for HIV and TB	
2015		-E-pocket book for hospital care for children	
